# Altered integration of speech and gesture in children with autism spectrum disorders

**DOI:** 10.1002/brb3.81

**Published:** 2012-08-10

**Authors:** Amy L Hubbard, Kristin McNealy, Ashley A Scott-Van Zeeland, Daniel E Callan, Susan Y Bookheimer, Mirella Dapretto

**Affiliations:** 1 Ahmanson-Lovelace Brain Mapping Center, University of CaliforniaLos Angeles, California; 2 Department of Modern Languages, Carnegie Mellon UniversityPittsburgh, Pennsylvania; 3 Department of Computational Brain Imaging, Neural Information Analysis LaboratoriesKyoto, Japan; 4 Neuroscience Interdepartmental Program, University of CaliforniaLos Angeles, California; 5 Department of Psychiatry and Biobehavioral Sciences, University of CaliforniaLos Angeles, California

**Keywords:** Autism spectrum disorders, fMRI, gesture, language, superior temporal gyrus

## Abstract

The presence of gesture during speech has been shown to impact perception, comprehension, learning, and memory in normal adults and typically developing children. In neurotypical individuals, the impact of viewing co-speech gestures representing an object and/or action (i.e., iconic gesture) or speech rhythm (i.e., beat gesture) has also been observed at the neural level. Yet, despite growing evidence of delayed gesture development in children with autism spectrum disorders (ASD), few studies have examined how the brain processes multimodal communicative cues occurring during everyday communication in individuals with ASD. Here, we used a previously validated functional magnetic resonance imaging (fMRI) paradigm to examine the neural processing of co-speech beat gesture in children with ASD and matched controls. Consistent with prior observations in adults, typically developing children showed increased responses in right superior temporal gyrus and sulcus while listening to speech accompanied by beat gesture. Children with ASD, however, exhibited no significant modulatory effects in secondary auditory cortices for the presence of co-speech beat gesture. Rather, relative to their typically developing counterparts, children with ASD showed significantly greater activity in visual cortex while listening to speech accompanied by beat gesture. Importantly, the severity of their socio-communicative impairments correlated with activity in this region, such that the more impaired children demonstrated the greatest activity in visual areas while viewing co-speech beat gesture. These findings suggest that although the typically developing brain recognizes beat gesture as communicative and successfully integrates it with co-occurring speech, information from multiple sensory modalities is not effectively integrated during social communication in the autistic brain.

## Introduction

Autism spectrum disorders (ASD) are a class of neurodevelopmental disorders characterized by impairments in social interaction and communication, as well as repetitive or stereotyped behaviors (American Psychiatric Association [*DSM-IV-TR*] 2000). In addition to these characteristic diagnostic criteria, individuals with ASD exhibit impairments in a host of higher cognitive functions, such as theory of mind, empathy, language, and imitation (for review, see Klin et al. 2002; Minshew and Williams 2007; Oberman and Ramachandran 2007). Due to the developmental trajectory of these cognitive skills, early diagnosis and intervention are paramount to redirect the course of atypical development associated with ASD. Language delay is one of the earliest observed symptoms of an ASD, and language ability is one of the most accurate predictors of future outcomes (Venter et al. 1992). Recently, it has been shown that delay in gesture development (i.e., pointing) is also observed in conjunction with delays in language development (Trillingsgaard et al. 2005; Colgan et al. 2006; Mitchell et al. 2006; Wetherby et al. 2007; Luyster et al. 2008; Sowden et al. 2008) – potentially even in advance of discernable language delay (Mitchell et al. 2006) – and that gesture impairments persist into later childhood years (Camaioni et al. 2003). With regard to gesture perception, a recent behavioral study (Klin et al. 2009) showed that children with autism – unlike typically developing (TD) children and developmentally delayed children – demonstrated no preference for speech-linked biological motion. Surprisingly, however, there is currently no information on the neural correlates of gesture processing in children with autism.

Co-speech gesture (i.e., gesture produced during speech communication) has been extensively studied in TD children. Infants at the one-word stage have been found to both use and understand gesture (Morford and Goldin-Meadow 1992), and gesture use is a reliable predictor of single-word and two-word acquisition (Iverson and Goldin-Meadow 2005), as well as more complex speech constructions (Özçalışkan and Goldin-Meadow 2005). Later in development, a child's gesture use becomes more complex (e.g., indicating objects, highlighting speech intonation, and representing metaphorical thinking; McNeill 1992) and can facilitate learning (Breckinridge-Church and Goldin-Meadow 1986; Goldin-Meadow and Sandhofer 1999; Goldin-Meadow and Singer 2003; Goldin-Meadow and Wagner 2005). Furthermore, gesture use by the child learner has been shown to aide information retention (Cook et al. 2008), and gesture use by the teacher has been shown to aide instruction (Goldin-Meadow and Singer 1999; Singer and Goldin-Meadow 2005).

Informed by the vast body of research highlighting abnormal development of gesture use in children with ASD and the importance of gesture in typical development, here we used functional magnetic resonance imaging (fMRI) to investigate neural responses to beat gesture in a group of children with ASD and an age-, IQ-, and gender-matched group of TD children. It has recently been shown that speech accompanying gestures mimicking objects or actions (i.e., iconic gestures; McNeill 1992) that facilitated comprehension in neurotypical individuals failed to facilitate comprehension in individuals with ASD (Silverman et al. 2010). In this study, we sought to investigate gesture and speech integration in the context of gesture that does not communicate semantic information. Furthermore, focusing on beat gesture – a type of co-speech gesture marking speech intonation and rhythm – may be particularly interesting given the extensive evidence of prosodic deficits in individuals with autism (Pronovost et al. 1966; Baltaxe and Simmons 1975, 1977; Paul 1987; Baltaxe and D'Angiola 1992; Shriberg et al. 2001; Rutherford et al. 2002; McCann and Peppe 2003; Kujala et al. 2005).

In light of their communicative deficits and abnormal gesture development, we predicted that children with ASD would utilize different neural resources to process co-speech beat gesture than their TD counterparts. More specifically, we expected TD children to process beat gesture and speech similarly to normal adults (Holle et al. 2008; Hubbard et al. 2009), showing increased responses not only in visual and motor areas but also in speech processing regions such as the superior temporal gyrus (STG). In contrast, we hypothesized that children with ASD would not demonstrate this modulatory effect in language areas while viewing co-speech beat gesture.

## Methods

### Participants

Thirteen high-functioning children with ASD and 13 TD children were recruited through referrals from the UCLA Autism Clinic, through flyers posted in the Los Angeles area, as well as from a pool of subjects who had previously participated in other research studies at UCLA. Inclusion criteria for the ASD group included the following: (1) a clinical diagnosis of ASD confirmed using the Autism Diagnostic Observation Schedule-Generic (ADOS-G; Lord et al. 2000) and the Autism Diagnostic Observation Interview-Revised (ADI-R; Lord et al. 1994), (2) no other known neurological disorders, (3) no structural brain abnormalities, and (4) fluent verbal abilities. Typically developing subjects had no history of medical, psychiatric, or neurological disorders according to parental report. All subjects were healthy, right-handed, and native English speakers who neither spoke nor understood American Sign Language (ASL). Data from three participants in the ASD group and three participants in the TD group were excluded due to severe motion artifacts. Data were analyzed for 10 children with ASD (10 males; 13.1 ± 2.1 years of age) and for 10 TD children (10 males; 12.1 ± 1.6 years of age). Age, IQ, and motion parameters did not significantly differ between our final ASD and TD samples. Three children with ASD were taking medication at the time of the fMRI scan; more specifically, one participant was taking an atypical antipsychotic, and two were taking a psychostimulant together with an antipsychotic. Table 1 shows the mean Verbal, Performance, and Full-Scale IQ (assessed by the Wechsler Intelligence Scale for Children – Third Edition or the Wechsler Abbreviated Scale of Intelligence; Wechsler 1991, 1999) for both ASD and TD groups. Also shown in this table are the mean scores on the communication and social subscales of the ADOS-G and the Social Responsiveness Scale (SRS; Constantino et al. 2000, 2003).

**Table 1:** Participants' characteristics

Characteristics	TD (mean ± SD)	ASD (mean ± SD)	Group comparison (*P-*value)
Chronological age (years)	12 ± 1.6	13 ± 2.1	0.36
Verbal IQ	115 ± 13	107 ± 16	0.46
Performance IQ	111 ± 7	113 ± 15	0.63
Full-scale IQ	116 ± 10	110 ± 14	0.52
ADOS communication subscale	NA	3.9 ± 2	NA
ADOS social subscale	NA	7.9 ± 3	NA
SRS	NA	117 ± 23	NA

TD, typically developing; ADOS, Autism Diagnostic Observation Schedule; SRS, Social Responsiveness Scale.

### Stimuli and activation paradigm

Stimuli were the same as those we previously used in a study on the neural correlates of beat gesture in neurotypical adults (Hubbard et al. 2009). All video segments composing the stimuli were culled from 2 h of spontaneous speech recorded in a naturalistic setting (i.e., the kitchen of a house). The recording featured a female native speaker of North American English who was naïve to the purpose of the recording. A set of questions relevant to the speaker's life and experiences was prepared prior to the recording. During the recording, the speaker was asked to stand in the kitchen and answer questions posed to her by the experimenter in the adjacent room. Great care was taken to remove speech articulators and other indices of fundamental frequency in an uncontrived, ecologically valid manner. The illusion of a cupboard occluding the speaker's face was created by affixing a piece of plywood (stained to match the wood in the kitchen) to the wall above the stove. Utilizing this naturally produced sample of speech and gesture (i.e., unscripted and not acted) enabled us to construct stimuli that closely resemble real-world use of conversational speech and gesture.

The recording was produced using a Sony DCR-HC21 Mini DV Handycam Camcorder secured on a tripod and tilted downward so that only the speaker's lower neck, torso area, and upper legs were visible. The speaker moved freely and expressed herself in a natural, conversational style throughout the recording. Importantly, although her head was behind the plywood board, her gaze was free to shift from the board directly in front of her to the observer sitting on the couch in the adjacent room.

**Figure 1:**
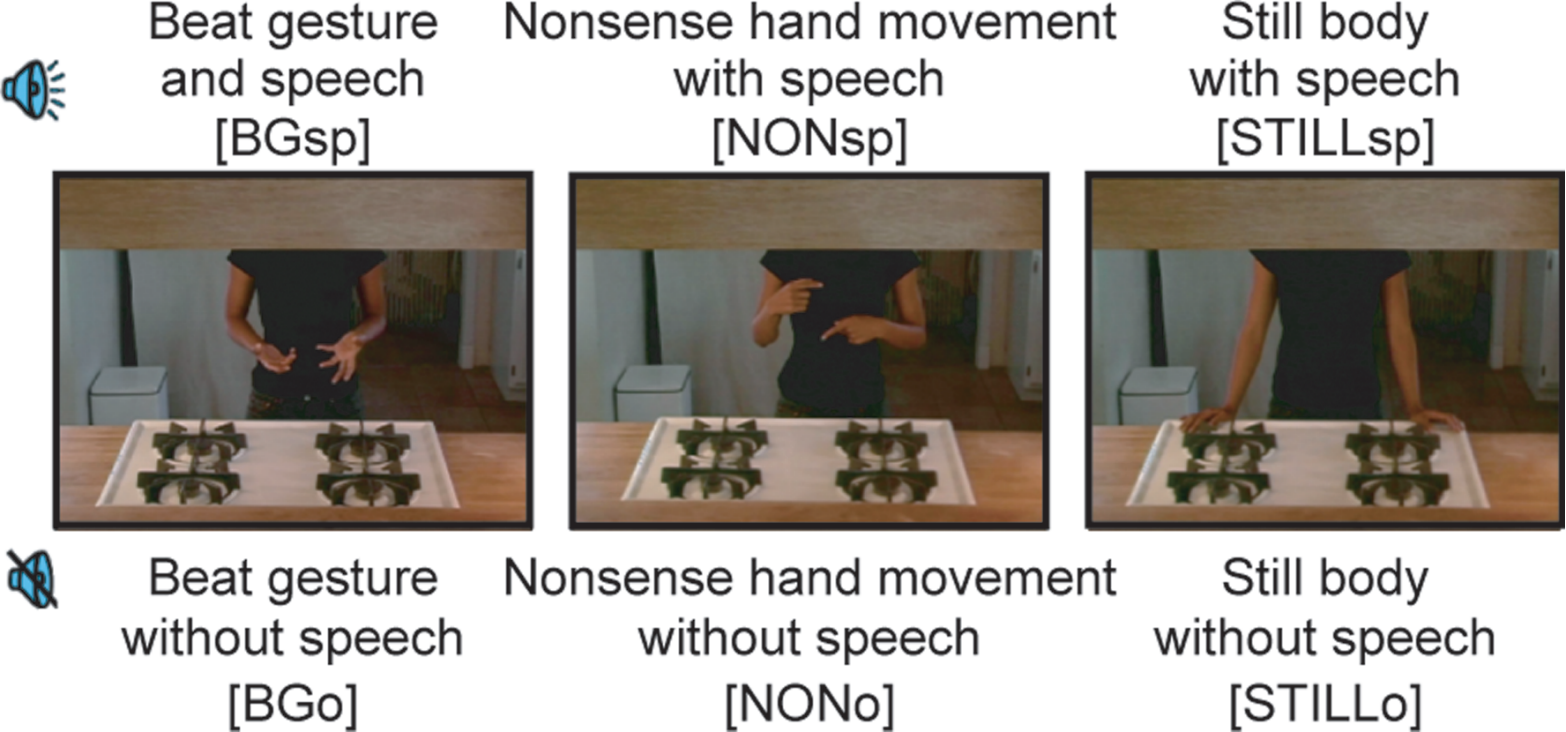
Experimental paradigm. There were six conditions, obtained by crossing movement type (beat gesture, nonsense hand movement, and still frame) by speech (present or absent). In the actual experiment, blocks were presented in pseudorandom orders counterbalanced across subjects.

Following the spontaneous speech recording, preplanned recordings that would comprise the still body and nonsense hand movement conditions were made. To create the image for the still body condition, the speaker was recorded as she stood motionless. Next, 12 picture sequences were affixed to the plywood board in front of the speaker's face, therefore, hidden from the viewpoint of the video camera. The pictures depicted movements that represent words in ASL but which lack obvious iconic meaning to nonsigners (see Fig. 1). The speaker, who neither spoke nor understood ASL, produced each set of movements one time (she neither saw nor practiced the movements in advance of the single-take recording). There were no words written on the pictures, and the speaker did not talk while producing the hand movements. We chose to use (noniconic) ASL hand shapes and movements in the control movement condition in order to include a set of hand movements that were produced in the same physical space as beat gesture (i.e., generally in front of the torso), varied in usage of one or both hands, and lacked rhythmic and communicative qualities (when produced by an ASL-naïve speaker).

Videos were captured with a Sony Mini DV GV-D900 and imported using Macintosh OSX and iMovie. Final Cut Pro HD 4.5 was used to cut and export twenty-four 18-sec segments of speech with beat gesture to .avi movie files. As the segments were selected from 2 h of free-flowing speech with gesture, inclusion or exclusion of gesture type could be controlled by cropping. That is, it was possible to eliminate movements that communicated consistent semantic information in the absence of speech by beginning an 18-sec segment after that gesture had occurred. Thus, the gesture in the final stimuli was tightly linked to speech prosody but did not convey semantic information when viewed without the originally co-occurring speech.

As the benefits of segregating gesture into strict categories has recently come under scrutiny (McNeill 2005), in order to maintain ecological validity, beat gesture (i.e., rhythmic gesture) was not limited to flicks of the hand for the purposes of this study (see Hubbard et al. 2009 for discussion). In the absence of an established method for determining the direct relationship between speech and gesture timing in free-flowing speech, we retained 18-sec segments of rhythmic gesture and speech that did not contain highly iconic gestures. A group of eight viewers (who were not subjects in the study) reported that semantic information could not be discerned by viewing the video segments in the absence of speech. Because the 24 speech segments used in our prior study in adults (Hubbard et al. 2009) varied in complexity, a subset of 12 segments was selected for this study based on appropriateness for a younger audience. Additionally, one 18-sec segment with a still frame of the speaker's body and six segments of ASL-based movements, consisting of 65 different signs, were selected. The selected ASL movements were noniconic, and a group of eight viewers (who did not participate in the study) again confirmed that the movements did not elicit semantic information.

All participants viewed a total of 18 videos in a single 6-min 30-sec run. Pseudorandomized video blocks involved six conditions, obtained by crossing movement type (beat gesture, nonsense hand movement, and still frame) by speech (present or absent). The 12 age-appropriate segments of beat gesture and speech were used in the “beat gesture with speech” condition (as originally recorded) and in the “beat gesture without speech” condition (where the audio was removed; see Fig. 3). The six ASL-based segments were used in the “nonsense hand movement without speech” condition (as originally recorded) and in the “nonsense hand movement with speech” condition (where they were paired with speech from the former 12 segments that were originally accompanied by beat gesture). Finally, the motionless recording of the speaker was used in the “still frame without speech” condition, used as baseline, and in the “still frame with speech” condition (where they were paired with speech from the 12 segments originally accompanied by beat gesture). One 18-sec segment was shown per block, and thus, blocks were 18-sec long, with a 3-sec blank screen separating segments.

The RMS energy of the audio segments was adjusted to be identical across stimuli. To prevent specific item effects (in terms of speech content), stimuli were counter-balanced across subjects such that one subject might hear and see segment #1 with the original beat gesture and speech, another subject might hear the speech of segment #1 while viewing one of the segments of nonsense hand movement, and yet another subject might hear the speech of segment #1 while viewing the still frame. For each subject, any part (speech and/or body movements) of the original 12-beat gesture segments and six nonsense hand movement segments occurred exactly one time. The order of presentation of the video segments was randomized subject to the constraints that there would be no serial occurrence of (i) two identical conditions, (ii) three segments with speech, or (iii) three segments without speech. Each subject in each group viewed a different randomization order of the video sequences.

### Data acquisition

Prior to entering the MRI suite, subjects received a short introduction to the task. They were shown a still picture of the video and told that the speaker, whose head was blocked by a cupboard in the kitchen, was talking to a person in the adjacent room. They were told that the speaker would sometimes be moving and talking, or be still and not talk, and that she would be talking about two topics (i.e., teaching surfing and building houses). To maintain the subjects' attention during the entire scan, subjects were advised that they would be given a postscan test on what they saw and heard. An abbreviated version of this description was also read to subjects, while they were on the scanner bed immediately prior to the fMRI scan.

Images were acquired using a Siemens Allegra 3 Tesla head-only MRI scanner in the UCLA Ahmanson-Lovelace Brain Mapping Center. A two-dimensional spin-echo image (repetition time [TR] = 4000 msec, echo time [TE] = 40 msec, matrix size 256 by 256, 4-mm thick, 1-mm gap) was acquired in the sagittal plane to allow prescription of the slices to be obtained in the remaining scans. For each participant, a high-resolution structural T2-weighted echo-planar imaging (EPI) volume (spin-echo, TR = 5000 msec, TE = 33 msec, matrix size 128 by 128, FOV = 20 cm, 36 slices, 1.56-mm in-plane resolution, 3-mm thick) was acquired coplanar with the functional scans to allow for spatial registration of each subject's data into a common space. During the gesture task, one functional whole-brain scan lasting 6 min and 30 sec was acquired (128 images, EPI gradient-echo, TR = 3000 msec, TE = 25 msec, flip angle = 90°, matrix size = 64 by 64).

Visual and auditory stimuli were presented to the subject using magnet-compatible three-dimensional goggles and headphones under computer control. The goggles, created by Resonance Technologies, Inc. (Northridge, CA), contain two miniature television sets with full 512 × 512 resolution that are placed inside a small goggle (similar to ski goggles) and worn by placing them directly over the participant's eyes. The audiovisual stimuli were presented using full view in Real Player in order to ensure that subjects saw no words, numbers, or time bars while viewing the stimuli.

### Data analysis

Following image conversion, the functional data were analyzed using Statistical Parametric Mapping 5 (SPM5; http://www.fil.ion.ucl.ac.uk/spm/software/spm5/). Functional images for each participant were realigned to correct for head motion, normalized into Montreal Neurological Institute (MNI) space (Mazziotta et al. 2001), and smoothed with a 6-mm Gaussian kernel. For each subject, condition effects were estimated according to the General Linear Model using a 6-sec delay boxcar reference function, high-pass filtering, and no global scaling. The still frame condition was implicitly modeled as baseline. The resulting contrast images were entered into second-level analyses using random effect models to allow for inferences to be made at the population level (Friston et al. 1999). For each group (ASD and TD), separate one-sample *t*-tests were implemented for each condition relative to baseline and between conditions (e.g., “beat gesture with speech” vs. “nonsense hand movement with speech”). Two-sample *t*-tests were used to examine between-group differences in each condition and in relevant between-condition contrasts. These analyses were performed within regions, where reliable activity was detected in either group during the “beat gesture with speech” condition (*P* < 0.05, cluster corrected for multiple comparisons). Further Region of Interest (ROI) analyses were conducted within areas where significant between-group differences were observed for this contrast. Finally, regression analyses were conducted in the ASD group using the subjects' scores on the SRS (Constantino et al. 2000, 2003) and the social and communication subscales of the ADOS-G (Lord et al. 2000) to investigate the relationship between symptom severity in the social and communicative domains and activity observed for the “beat gesture with speech” contrast (vs. “beat gesture with still frame”).

Activation maps for all within-group comparisons and regression analyses were thresholded at *P* < 0.005 for magnitude, with whole-volume correction for multiple comparisons applied at the cluster level (*P* < 0.05). Activation maps for between-group analyses were thresholded at *P* < 0.01 for magnitude, with whole-volume correction for multiple comparisons applied at the cluster level (*P* < 0.05). The SPM toolbox MarsBaR (Brett et al. 2002a,b) and MarsBaR AAL ROI package (Brett et al. 2002a,b) were used to extract parameter estimates for each participant from ROIs. Cluster size and coordinates for peaks of activity for all contrasts of interest are presented in Tables 2–5.

**Table 2:** Significant activity observed in typically developing children for each contrast of interest

Anatomical region	Peak (MNI; mm)			Extent (voxels)	Max (*t*)	Cluster (*P*)
*Still frame with speech* versus *Still frame without speech (STILLsp>STILLo; baseline)*						
L superior temporal gyrus and sulcus, middle temporal gyrus, Heschl's gyrus, and Rolandic operculum	−52	−20	6	3096	17.8	<0.001
	−60	−8	−2		12.67	
R superior temporal gyrus, sulcus, and pole, middle temporal pole, and Heschl's gyrus	64	−8	2	1508	11.20	<0.001
	48	−26	8		9.69	
L precuneus	−6	−60	24	561	9.64	<0.001
L gyrus rectus and bilateral medial orbital gyri	0	34	−18	329	9.39	<0.001
L posterior middle temporal gyrus and angular gyrus	−48	−68	24	200	4.95	<0.004
L medial superior frontal gyrus	−4	56	12	190	5.11	<0.006
*Beat gesture with speech* versus *Still frame without speech (BGsp>STILLo; baseline)*						
L superior temporal gyrus and sulcus, middle temporal gyrus,	−44	−26	4	4687	15.40	<0.001
Heschl's gyrus, middle and inferior occipital gyri, and angular gyrus	−60	−16	4		12.68	
R superior temporal gyrus, sulcus, and pole, middle temporal gyrus and pole, and Heschl's gyrus	52	−4	−14	2606	11.53	<0.001
	66	−20	10		9.66	
L superior and middle frontal gyri	−10	36	48	634	10.00	<0.001
L gyrus rectus	0	38	−20	374	7.88	<0.001
R middle temporal gyrus and middle occipital gyrus	48	−62	2	331	5.57	<0.001
R cerebellum	14	−44	−38	160	5.61	<0.019
*Nonsense hand movement with speech* versus *Still frame without speech (NONsp>STILLo; baseline)*						
L superior temporal gyrus and sulcus, middle temporal gyrus and pole, Heschl's gyrus, Rolandic operculum, and middle and inferior occipital gyri	−58	−18	6	6648	20.21	<0.001
	−60	−6	0		17.10	
R superior temporal syrus and sulcus, Heschl's gyrus, posterior middle and inferior temporal gyri, and middle and inferior occipital gyri	46	−72	0	3573	15.10	<0.001
	58	−16	6		11.04	
L superior middle frontal gyrus	−4	58	24	256	6.68	<0.001
L inferior orbital frontal gyrus	−40	26	−6	247	7.62	<0.001
L fusiform gyrus	−40	−48	−18	242	7.76	<0.001
*Beat gesture without speech* versus *Still frame without speech (BGo>STILLo; baseline)*						
L middle occipital gyrus, middle and inferior temporal gyri, and angular gyrus, bilateral middle orbital frontal gyri, and bilateral gyrus rectus	−48	−76	4	1204	11.37	<0.001
	−6	46	−8	537	8.91	<0.001
L medial superior and superior frontal gyri and bilateral anterior cingulate gyrus	−2	56	12	587	7.12	<0.001
R inferior and middle occipital gyri, and posterior middle and inferior temporal gyri	46	−74	−2	507	7.11	<0.001
L inferior orbital frontal gyrus	−36	32	−12	234	6.68	<0.001
R middle temporal gyrus and pole and inferior temporal gyrus	56	10	−24	224	4.36	<0.001
Bilateral precuneus, L posterior cingulum	−4	−50	20	166	5.75	<0.007
R cuneus, superior occipital gyrus, and calcarine gyrus	18	−98	8	126	6.55	<0.036
*Nonsense hand movement without speech* versus *Still frame without speech (NONo>STILLo; baseline)*						
R posterior superior, middle and inferior temporal gyri, middle and inferior occipital gyri	36	−66	8	1615	10.72	<0.001
L superior, middle, and inferior orbitofrontal gyri	−44	34	−10	514	9.84	<0.001
L posterior middle and inferior temporal gyri, middle and inferior occipital gyri, angular gyrus	−46	−70	4	1630	9.75	<0.001
L inferior and superior parietal gyri, postcentral gyrus, supramarginal gyrus	−28	−40	56	671	9.57	<0.001
R precentral gyrus, postcentral gyrus, supramarginal gyrus	58	−16	44	212	8.81	<0.001
L supramarginal gyrus, inferior parietal gyrus	−58	−32	26	395	7.89	<0.001
R postcentral gyrus	34	−32	42	434	7.34	<0.001
Bilateral gyrus rectus	−6	34	−20	147	6.69	<0.013
*Beat gesture with speech* versus *Still frame with speech (BGsp>STILLsp)*						
R superior temporal gyrus and sulcus	42	−56	4	738	6.34	<0.001
R posterior middle and inferior temporal gyri, middle and inferior occipital gyri	44	−74	−2		5.90	
R posterior middle temporal gyrus, middle and inferior occipital gyri	−50	−64	6	573	6.28	<0.001
*Nonsense hand movement with speech* versus *Still frame with speech (NONsp>STILLsp)*						
R posterior middle and inferior temporal gyri, middle and inferior occipital gyri	48	−72	−2	1843	12.29	<0.001
L posterior middle and inferior temporal gyri, middle and inferior occipital gyri	−52	−76	2	1508	11.11	<0.001
*Beat gesture with speech* versus *Nonsense hand movement with speech (BGsp>NONsp)*						
No clusters survived correction for multiple comparisons						

*x*, *y*, and *z* = the MNI coordinates (mm) corresponding to the left–right, anterior–posterior, and inferior–superior axes, respectively; *t*, the highest *t*-score within a region; thresholded at *t* > 3.36 (*P* < 0.005); corrected for multiple comparisons at the cluster level (*P* < 0.05).

**Table 3:** Significant activity observed in children with ASD for each contrast of interest

Anatomical region	Peak (MNI; mm)			Extent (voxels)	Max (*t*)	Cluster (*P*)
*Still frame with speech* versus *Still frame without speech (STILLsp>STILLo; baseline)*						
L superior temporal gyrus, sulcus, and pole, middle temporal gyrus and pole, Heschl's gyrus, and Rolandic operculum	−50	−18	8	2509	19.1	<0.001
	−56	−10	2		10.7	
R superior temporal gyrus and sulcus, middle temporal gyrus, Heschl's gyrus, and Rolandic operculum	52	−8	−2	2003	11.9	<0.001
	58	0	−6		10.6	
L inferior frontal gyrus (pars triangularis and pars opercularis)	−46	22	16	254	4.94	<0.006
*Beat gesture with speech* versus *Still frame without speech (BGsp>STILLo; baseline)*						
L thalamus, middle temporal gyrus, superior temporal gyrus, Heschl's gyrus, and Rolandic operculum	−12	−32	4	3159	9.18	<0.001
	−50	−22	10		8.54	
R superior temporal gyrus and sulcus, middle temporal gyrus, and Heschl's gyrus	58	−6	−6	1854	11.05	<0.001
R middle and inferior temporal gyri and middle and inferior occipital gyri	48	−74	−2	503	8.14	<0.001
L middle and inferior occipital gyri and middle temporal gyrus	−34	−70	2	894	7.45	<0.001
R middle occipital gyrus and calcarine gyrus	30	−90	6	132	6.86	<0.004
L inferior frontal gyrus (pars triangularis)	−38	26	12	105	7.09	<0.019
R hippocampus	32	−8	−18	104	7.02	<0.021
*Nonsense hand movement with speech* versus *Still frame without speech (NONsp>STILLo; baseline)*						
R superior temporal gyrus and sulcus, Heschl's gyrus, Rolandic operculum, posterior middle and inferior temporal gyri, and middle and inferior occipital gyri	−62	−16	4	4734	13.2	<0.001
	−52	−68	−4		10.4	
R superior temporal gyrus and sulcus, middle temporal gyrus, and Heschl's gyrus	48	−26	4	1950	9.82	<0.001
R middle and inferior temporal gyri and middle and inferior occipical gyri	52	−72	4	475	8.82	<0.001
L anterior cingulum and superior medial frontal gyrus	−6	46	10	157	4.85	<0.002
L postcentral gyrus	−34	−40	62	114	7.66	<0.020
*Beat gesture without speech* versus *Still frame without speech (BGo>STILLo; baseline)*						
R posterior middle temporal gyrus, and middle and inferior occipital gyri	50	−68	2	207	6.54	<0.001
L posterior middle temporal gyrus, and middle occipital gyrus	−52	−74	2	81	5.71	<0.009
*Nonsense hand movement without speech* versus *Still frame without speech (NONsp>STILLsp; baseline)*						
L middle temporal gyrus	−64	−16	−16	109	15.7	<0.001
L inferior frontal gyrus (pars triangularis)	−44	24	12	132	9.82	<0.001
L hippocampus	−34	−16	−22	178	9.08	<0.001
L precuneus	−2	−38	68	136	8.77	<0.001
R hippocampus	32	−10	−22	218	8.13	<0.001
R postcentral gyrus, inferior parietal gyrus	22	−36	54	216	7.85	<0.001
L precentral gyrus, middle frontal gyrus	−32	−10	50	262	7.6	<0.001
Bilateral gyrus rectus	2	40	−16	751	7.41	<0.001
R posterior middle and inferior temporal gyri, middle and inferior occipital gyri	38	−80	6	817	6.82	<0.001
L inferior temporal gyrus	−56	−46	−10	80	6.82	<0.001
L cerebellum, middle and inferior occipital gyri	−36	−76	−26	908	6.66	<0.001
L superior supplementary motor area	−12	−8	72	66	6.1	<0.027
L thalamus	−6	−20	16	113	5.95	<0.001
*Beat gesture with speech* versus *Still frame with speech (BGsp>STILLsp)*						
L posterior middle and inferior temporal gyri, and middle and inferior occipital gyri	−36	−72	6	833	8.27	<0.001
R posterior middle and inferior temporal gyri, and middle and inferior occipital gyri	50	−72	0	774	17.7	<0.001
*Nonsense hand movement with speech* versus *Still frame with speech (NONsp>STILLsp; baseline)*						
L posterior middle and inferior temporal gyri, middle and inferior occipital gyri	−38	−68	0	788	8.54	<0.001
R posterior middle and inferior temporal gyri, middle and inferior occipital gyri	42	−72	−2	595	7.73	<0.001
*Beat gesture with speech* versus *Nonsense hand movement with speech (BGsp>NONsp)*						
No clusters survived correction for multiple comparisons						

*x*, *y*, and *z* = the MNI coordinates (mm) corresponding to the left–right, anterior–posterior, and inferior–superior axes, respectively; *t*, the highest *t*-score within a region; thresholded at *t* > 3.36 (*P* < 0.005); corrected for multiple comparisons at the cluster level (*P* < 0.05).

**Table 4:** Significant activity observed in between-group comparisons for contrasts of interest

	TD > ASD						ASD > TD					
		
Anatomical region	Peak (MNI; mm)			Extent (voxels)	Max (*t*)	Cluster (*P*)	Peak (MNI; mm)			Extent (voxels)	Max (*t*)	Cluster (*P*)
*Beat gesture with speech* versus *Still frame with speech (BGsp>STILLsp)*												
R STG/S and MTG	54	−30	4	205	4.10	<0.036						
R lingual gyrus, calcarine gyrus, and cuneus							16	−86	−2	196	5.05	<0.044
*Beat gesture without speech* versus *Still frame without speech (BGo>STILLo; baseline)*												
L middle and inferior temporal gyri and middle temporal pole	−42	4	−32	190	4.66	<0.011						
L inferior orbital frontal gyrus	−42	26	−14	178	4.05	<0.016						
R middle and inferior temporal gyri and middle temporal pole	54	4	−30	166	6.05	<0.022						
*Nonsense movement with speech* versus *Still frame without speech (NONsp>STILLo; baseline)*												
L superior and middle frontal gyri							−24	−2	60	285	4.46	<0.014

*x*, *y*, and *z* = the MNI coordinates (mm) corresponding to the left–right, anterior–posterior, and inferior–superior axes, respectively; *t*, the highest *t*-score within a region; thresholded at *t* > 2.55 (*P* < 0.01); corrected for multiple comparisons at the cluster level (*P* < 0.05).

**Table 5:** Areas showing positive correlations between scales measuring symptom severity in the ASD group and increased activity when viewing “beat gesture with speech” versus “still frame with speech”

Anatomical region	Peak (MNI; mm)			Extent (voxels)	Max (*t*)	Cluster (*P*)
*ADOS-G Communication Subscale*						
Left inferior and middle occipital gyri and posterior middle temporal gyrus	−34	−68	0	800	10.03	0.001
Right posterior inferior and middle temporal gyri, inferior and middle occipital gyri and calcarine and lingual gyri	50	−72	0	725	24.92	0.001
Right hippocampus	22	−28	−4	57	6.14	0.034
*ADOS-G Social Subscale*						
Left inferior and middle occipital gyri and posterior middle temporal gyrus	−34	−66	−2	961	9.86	0.001
Right posterior inferior and middle temporal gyri, inferior and middle occipital gyri and calcarine and lingual gyri	50	−68	2	782	20.21	0.001
Right hippocampus	22	−28	−4	91	6.17	0.002
*Social Responsiveness Scale (SRS)*						
Left inferior and middle occipital gyri and posterior middle temporal gyrus	−34	−66	−2	822	8.07	0.001
Right posterior inferior temporal gyrus and inferior and middle occipital gyri and lingual gyrus	50	−72	0	701	23.61	0.001
Right hippocampus	22	−28	−4	36	6.13	0.028

ADOS-G, Autism Diagnosis Observation Schedule-Generic (Lord et al. 2000); *x*, *y*, and *z* = the MNI coordinates (mm) corresponding to the left–right, anterior–posterior, and inferior–superior axes, respectively; *t*, the highest *t*-score within a region; thresholded at *t* > 3.36 (*P* < 0.005); corrected for multiple comparisons at the cluster level (*P* < 0.05).

## Results

### Whole-brain analyses

As shown in Tables 2 and 3, within-group contrasts revealed that both TD and ASD children activated similar language-relevant frontotemporal networks when responses for conditions involving the presentation of speech were compared with conditions without speech. Likewise, both group contrasts also showed increased activity in visual areas for conditions involving body movement versus conditions involving a still frame. The overall similar pattern of activity observed in each group across conditions suggests that both TD and ASD children attended to and processed the relevant features of our stimuli (but see below and Table 4 for between-group contrasts).

**Figure 2:**
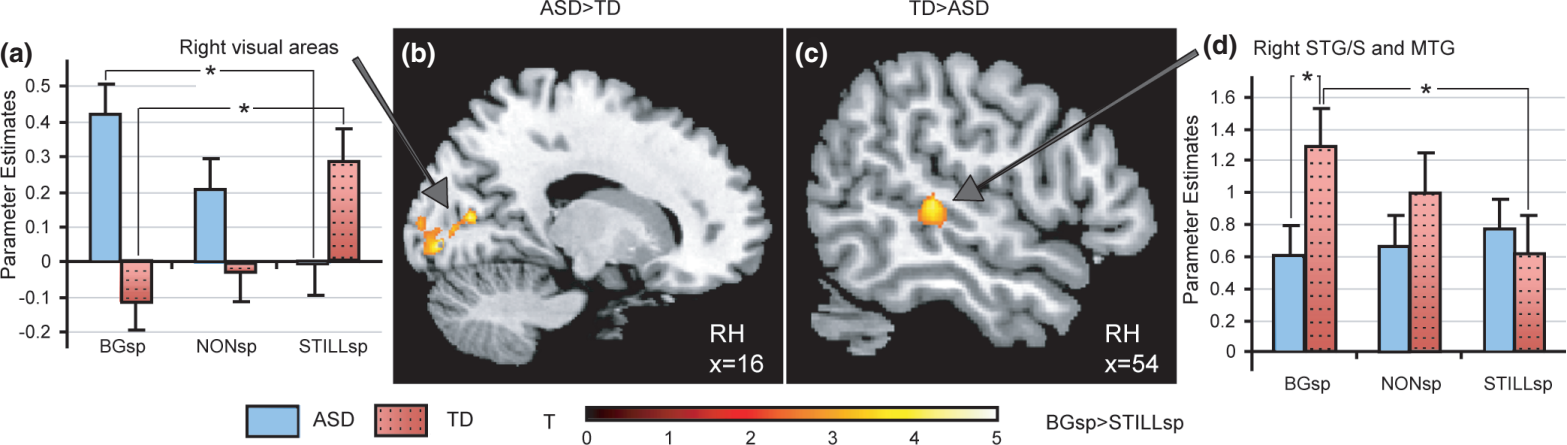
Differences in neural activity for ASD and TD groups related to processing “still frame with speech” and “beat gesture with speech.” Clusters depict areas of significantly greater activity while viewing “beat gesture with speech” as compared with viewing “still frame with speech” (b) ASD versus TD and in (c) TD versus ASD. Parameter estimates within the regions showing significantly greater activity in (a) ASD versus TD (maxima located at 16, −86, 2; MNI coordinates) and (d) TD versus ASD (maxima located at 54, −30, 4; MNI coordinates) while viewing “beat gesture with speech” as compared with viewing “still frame with speech.” Specific contrasts are depicted using the abbreviated condition names defined in Figure 1. Group activation maps were thresholded at *t* > 2.55 (*P* < 0.01) for magnitude, with correction for multiple comparisons at the cluster level (*P* < 0.05). Error bars equal standard error of the mean. RH, right hemisphere.

With regard to our primary contrast of interest – “beat gesture with speech” versus “still frame with speech” – both groups showed significantly greater activity in visual cortices (see Tables 2 and 3). However, in addition to the extensive increased activity observed in visual areas, significant activity was also observed in right posterior STG and sulcus (STG/S) for the TD group and in bilateral posterior middle and inferior temporal gyri for the ASD group. A direct between-group comparison for this contrast revealed significantly greater activity in TD than ASD children in the right STG/S and middle temporal gyrus (MTG), and greater activity in ASD than TD children in lingual gyrus, calcarine fissure, and cuneus (see Fig. 2b and c).

The significant between-group differences observed when speech was accompanied by beat gesture were not observed when speech was accompanied by nonsense hand movement. Within-group analyses for both the TD and ASD groups showed that bilateral middle and inferior occipital gyri as well as bilateral posterior middle and inferior temporal gyri were more active while viewing “nonsense hand movement with speech” (vs. “still frame with speech”; see Tables 2 and 3). Between-group analyses revealed no significant differences in viewing “nonsense hand movement with speech” versus “still frame with speech”.

### ROI analyses

To further examine the effect of co-speech beat gesture within language and visual processing regions in both TD children and children with ASD, we extracted the raw parameter estimates for each group from two ROIs defined as the 205-voxel cluster in right STG/S and MTG where significantly greater activity was observed for TD than ASD children and the 196-voxel cluster in visual areas where significantly greater activity was observed for ASD than TD children in the whole-brain analyses. The parameter estimates for the “beat gesture with speech, nonsense hand movements with speech,” and “speech with still frame” contrasts (vs. the “still frame without speech” baseline) were then entered into two separate 2 (Group) × 3 (Condition) repeated-measures analyses of variance (ANOVAs), one for each ROI. For the STG/S and MTG ROI, this analysis revealed a significant group by condition interaction, *F*(1,18) = 13.97, *P* < 0.005, which was qualified by significant between-group differences for “beat gesture with speech,” *F*(1,18) = 4.74, *P* < 0.05, and a lack of significant between-group differences for “nonsense hand movement with speech” or “still frame with speech” (*P*s > 0.14; see Fig. 2d). Furthermore, the TD group showed significantly greater activity in this ROI for speech accompanied by beat gesture versus speech accompanied by a still frame (*P* < 0.005; see Fig. 2d, red bars). In contrast, the ASD group showed equal responses in this region across all conditions, regardless of whether speech was accompanied by beat gesture, nonsense hand movements, or a still frame (*P*s > 0.32; see Fig. 2d, blue bars). Importantly, significantly greater responses to “beat gesture with speech” for the TD group (vs. the ASD group) were not limited to this specific portion of right STG, as the raw parameter estimates extracted from an anatomical ROI which included the entire right STG (Tzourio-Mazoyer et al. 2002) demonstrated the same significant between-group differences for viewing “beat gesture with speech.”

For the ROI encompassing the visual areas where the ASD group showed significantly greater activity than the TD group, the ANOVA also revealed a significant group by condition interaction, *F*(1,18) = 21.69, *P* < 0.001 (see Fig. 2a). More specifically, for the ASD group, activity in this ROI was significantly greater when viewing “beat gesture with speech” versus viewing a “still frame with speech” (*P* < 0.005; see Fig. 2a, blue bars). Interestingly, the TD group showed the opposite effect whereby responses for “still frame with speech” were significantly greater than for “beat gesture with speech” (*P* < 0.005; see Fig. 2a, red bars).

Given that three participants with ASD were taking medications at the time of the scan, we inspected their data to evaluate whether they may have impacted our results. Parameter estimates for these three participants fell well within the range observed for the participants who were not taking medications for all condition with the following exceptions. One of the two participants taking both a psychostimulant and an antipsychotic drug had the highest (i.e., a more “normative”) level of activity observed within the ASD group for “beat gesture with speech” within the STG/S ROI; in contrast, the participant taking an atypical antipsychotic had the lowest (i.e., more atypical) level of activity for this same contrast and ROI. The third participant who was also taking a psychostimulant and an antipsychotic drug had the lowest (i.e., more “normative”) level of activity for “beat gesture with speech” in the ROI encompassing the visual areas, where greater activity was observed in the ASD versus the TD group. All reported between-group differences held when these subjects were excluded from our ROI analyses.

### Regression analyses

**Figure 3:**
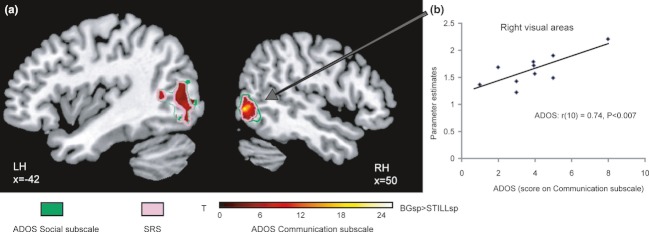
Activity in visual areas and symptom severity. (a) While viewing “beat gesture with speech” versus viewing “still frame with speech,” positive correlations were found in the ASD group between activity in bilateral visual areas (i.e., inferior and middle occipital gyri, lingual gyrus, calcarine gyrus, posterior inferior temporal gyrus, and posterior middle temporal gyrus) and scores on the social subscale of the ADOS (yellow), communication subscale of the ADOS (blue), both social and communication subscales of the ADOS (purple), and the SRS (red). (b) Positive correlation between scores on ADOS social subscale (yellow circles), ADOS communication subscale (blue triangles), and SRS (red diamonds) and parameter estimates of activity in visual areas for the contrast of “beat gesture with speech” versus “still frame with speech” (maxima for ADOS social subscale 50, −68, 2; ADOS communication subscale 50, −72, 0; and SRS 50, −72, 0; MNI coordinates). Group activation maps were thresholded at *t* > 3.36 (*P* < 0.005) for magnitude, with correction for multiple comparisons at the cluster level (*P* < 0.05). RH, right hemisphere. LH, left hemisphere.

To investigate the degree to which socio-communicative impairment might be linked to the neural processing of co-speech gesture, we examined the relationship between activity related to co-speech gesture processing and symptom severity, as indexed by children's scores on the ADOS-G (Lord et al. 2000) and the SRS (Constantino et al. 2000, 2003) in which higher scores indicate greater impairment. When contrasting the ASD participants' individual responses to “beat gesture with speech” versus “still frame with speech,” we found reliable positive correlations between activity in bilateral visual areas (e.g., occipital gyri and posterior temporal gyri; see Table 5, Fig. 3a and b) and children's scores on the social subscale of the ADOS-G (see Fig. 3a, yellow; Fig. 3b, yellow dots), the communication subscale of the ADOS-G (see Fig. 3a, blue; Fig. 3b, blue triangles), and the SRS (see Fig. 3a, red; Fig. 3b, red diamonds). That is, the greater the symptom severity on all these measures, the greater the activity observed in these regions of visual cortex. Finally, we examined whether age modulated activity in the STG/S in response to “beat gesture with speech” (vs. “still frame with speech”) and found no significant correlations with age in either group.

## Discussion

Here, we sought to investigate how children with ASD integrate multimodal cues during social communication. In light of the linguistic and socio-communicative impairments that characterize this disorder, we hypothesized that children with ASD would demonstrate abnormal neural responses while viewing co-speech beat gesture. Indeed, our results confirmed that children with ASD recruited different neural networks during the processing of co-speech beat gesture than age- and IQ-matched TD counterparts.

Similar to what has been observed in neurotypical adults (Holle et al. 2008; Hubbard et al. 2009), the TD children in our study showed increased activity in STG/S while viewing co-speech gesture. In contrast, the children with ASD did not show significant increases in activity within these regions specific to the presence of co-speech beat gesture. Furthermore confirming this observation, direct group comparisons showed that STG/S was significantly more active in response to the presence of co-speech beat gesture in TD children than in children with ASD. Rather, the direct group comparisons revealed that children with ASD showed significantly greater activity than TD children within visual areas when processing co-speech beat gesture. Interestingly, activity in these visual areas was found to positively correlate with symptom severity as indexed by both the ADOS-G and SRS. Between-group comparisons of STG/S activity in response to viewing co-speech beat gesture – observed both in neurotypical adults and in TD children – may represent the integration of multimodal speech cues. Thus, for children with ASD, the observation that co-speech beat gesture has a modulatory effect on visual cortices (and that this effect becomes greater as a function of symptom severity) instead of on STG/S suggests that the auditory and visual aspects of the stimuli are being processed somewhat independently. Taken together, these findings suggest that children with ASD are not effectively integrating information from multiple sensory modalities during social communication.

Although there are similarities between the responses we observed in this sample of TD children and those we previously observed in normal adults (Hubbard et al. 2009) for viewing co-speech beat gesture, there were also a number of differences. Neurotypical adults demonstrate greater activity in right anterior STG for the contrast of beat gesture with speech versus nonsense hand movement with speech (Hubbard et al. 2009); in TD children, however, significant differences for this contrast were observed only at liberal thresholds. Additionally, unlike neurotypical adults, TD children did not show increases in motor cortex in response to viewing co-speech beat gesture, and STG/S responses to co-speech beat gesture were limited to the right hemisphere (whereas responses were bilateral in normal adults). This decreased sensitivity in TD children may perhaps reflect developmental differences in multimodal speech perception. For example, in a seminal study on audiovisual speech perception (McGurk and MacDonald 1976), only 52% of TD children ages 7–8 years old were shown to be impacted by the presence of contradictory audiovisual speech cues. Future studies directly comparing children and adults are needed to further characterize developmental changes in the neural basis of multimodal speech perception.

In the case of children with ASD, increases in neural activity over that observed in TD controls is often interpreted as reflecting a compensatory strategy. For example, in Wang et al. (2006), increased activity for children with ASD (within regions recruited by TD controls) was suggested to reflect more effortful processing needed to complete the language processing task. Because there was no overt task in this study, it is unlikely that the additional activity we observed in visual areas reflects an explicit compensatory mechanism on the part of the children with ASD. Further support for this conclusion comes from an examination of areas in the brain, where activity was modulated by symptom severity. The visual areas identified in between-group analyses as showing stronger activity in the ASD children were the only areas in the brain where activity correlated with symptom severity: the more severe the ASD symptoms, the greater the activity in these visual areas. We therefore conclude that the abnormal activity observed in children with ASD in these regions is most likely indicative of a deficit in multisensory integration, observed most substantially (at both the neural and behavioral level) in children with the greatest symptom severity. The findings of Mongillo et al. (2008) lend further support to this interpretation as they found that SRS scores were negatively correlated with scores on the McGurk test – a test of auditory and visual speech integration (McGurk and MacDonald 1976). Thus, consistent with our results, greater symptom severity is associated with less evidence of multisensory integration.

The current findings – especially with regard to the positive correlation observed between symptom severity and neural activity in visual areas – are consistent with growing evidence of abnormal cortical connectivity in children with ASD (e.g., Kleinhans et al. 2008). It has been theorized that individuals with ASDs exhibit increased local connectivity, to the detriment of long-range connectivity (for review, see Minshew and Williams 2007). For example, several studies have identified decreased connectivity between visual and frontal cortices (Villalobos et al. 2005; Koshino et al. 2008), and other studies have found increases in thalamocortical connectivity, hypothesized to compensate for reduced cortico-cortical connectivity (Mizuno et al. 2006). Also, highly relevant to the current findings are studies reporting abnormal low-level visual processing (Bertone et al. 2005), visual hypersensitivity (Ashwin et al. 2009), and/or low-level visual problems (Vandenbroucke et al. 2008) in individuals with ASD. In this study, audiovisual integration – which depends on the synthesis of information from primary visual and auditory cortices – may be disrupted as a result of abnormal cortico-cortical connectivity and/or a specific deficit in visual processing. Future studies are needed to address these competing accounts.

Finally, our findings are in line with considerable evidence suggesting specific deficits in integrating communicative cues in individuals with ASD (Williams et al. 2004; Mongillo et al. 2008; Whitehouse and Bishop 2008; Klin et al. 2009). Recently, Mongillo et al. (2008) found that for a group of children with ASD, deficits in audiovisual integration were more salient when stimuli involved audiovisual elements of human communication (i.e., faces and voices) versus nonhuman visual and auditory stimuli. Similarly, Whitehouse and Bishop (2008) showed that children with ASD responded less to repetitive speech sounds than to repetitive nonspeech sounds, although responses to both types of sounds were the same when children with ASD were explicitly instructed to attend to the sounds. Williams et al. (2004) also reported deficits in audiovisual integration of visual speech (i.e., the movements of lips, mouth, and tongue which produce speech) in children with ASD. Klin et al. (2009) observed that 2-year-olds with ASD were more likely than controls to attend to nonbiological motion than to human biological motion. Most recently, Silverman et al. (2010) reported differences in how neurotypical individuals and individuals with ASD utilize iconic co-speech gesture to aide comprehension. Namely, the presence of iconic gesture facilitated comprehension in neurotypical individuals, but did not facilitate comprehension in individuals with ASD. There is behavioral and neural evidence of a tight link between gesture and speech integration during speech processing in neurotypical individuals (Özyürek et al. 2007; Willems et al. 2007, 2008; Kelly et al. 2010). The abnormal neural responses we observed in children with ASD while listening to speech accompanied by beat gesture (i.e., audiovisual stimuli which have inherent communicative value) provide additional evidence of disrupted processing of communicative audiovisual cues even in high-functioning individuals with ASD.

Taken together, these findings highlight the importance of further examining how individuals with ASD process information that is directly relevant to social communication. In face-to-face communication, there is continuous information available from multiple sensory modalities (e.g., facial expression, tone of voice, and body posture). This study is only the first to investigate how cues conveyed by hand gesture may impact speech perception in individuals with ASD; there remains much to be explored with regard to how individuals with ASD process other types of communicative cues in real-world contexts. Further work in this area would not only contribute to our understanding of the communicative impairments seen in ASD but may also inform the design of future diagnostic tools and behavioral interventions.
